# Contribution of ESC DAPT guideline-endorsed high thrombotic risk features to long-term clinical outcomes among patients with and without high bleeding risk after PCI

**DOI:** 10.1186/s12872-020-01600-3

**Published:** 2020-07-01

**Authors:** Hao-Yu Wang, Ke-Fei Dou, Dong Yin, Dong Zhang, Run-Lin Gao, Yue-Jin Yang

**Affiliations:** 1 Department of Cardiology, Coronary Heart Disease Center, Fuwai Hospital, State Key Laboratory of Cardiovascular Disease, National Center for Cardiovascular Diseases, Chinese Academy of Medical Sciences and Peking Union Medical College, Beijing, 100037 China; 2 State Key Laboratory of Cardiovascular Disease, Beijing, 100037 China; 3 National Clinical Research Center for Cardiovascular Diseases, Beijing, 100037 China

**Keywords:** Drug-eluting stent, High thrombotic risk, High bleeding risk, Guidelines, Outcomes, Percutaneous coronary intervention

## Abstract

**Background:**

Whether the underlying risk of high bleeding risk (HBR) influences the relationship of high thrombotic risk (HTR) features with adverse events after drug-eluting stent implantation remains unclear. The purpose of this study was to evaluate (1) the prognostic effect of ESC guideline-endorsed HTR features on long-term clinical outcomes and (2) whether the outcomes of HTR versus non-HTR features vary by HBR status.

**Methods:**

Ten thousand one hundred sixty-seven consecutive patients who underwent percutaneous coronary intervention between January 2013 and December 2013 were prospectively enrolled in Fuwai PCI Registry. Patients who are at HTR were defined as: diffuse multivessel disease in diabetic patients, chronic kidney disease, at least three stents implanted, at least three stents lesions treated, bifurcation with two stents implanted, total stent length > 60 mm, or treatment of chronic total occlusion. The definition of HBR was based on the Academic Research Consortium for HBR criteria. The primary ischemic outcome was major adverse cardiac event (MACE), a composite of cardiac death, myocardial infarction, target vessel revascularization and stent thrombosis. The primary bleeding outcome was clinically relevant bleeding, defined according to Bleeding Academic Research Consortium (BARC) type 2, 3 or 5 bleeding.

**Results:**

With a 2.4-year median follow-up, 4430 patients (43.6%) having HTR experienced a significantly higher risk of MACE (hazard ratio [HR] _adjust_: 1.56, 95% confidence interval [CI]: 1.34–1.82; *P* < 0.001) and device-oriented composite endpoint (composite of cardiac death, target-vessel MI, and target lesion revascularization) (HR_adjust_: 1.52 [1.27–1.83]; *P* < 0.001), compared to those having non-HTR. The risk of clinically relevant bleeding did not differ between groups (HR_adjust_: 0.85 [0.66–1.08]; *P* = 0.174). Associations between HTR and adverse events were similar in HBR and non-HBR groups, without evidence of interaction (all P_interaction_ > 0.05); however, adverse event rates were highest among subjects with both HTR and HBR.

**Conclusions:**

ESC guideline-endorsed HTR was associated with significantly increased risk of MACE without any significant differences in clinically relevant bleeding. The presence of HBR does not emerge as a modifier of cardiovascular risk for patients at HTR, suggesting more potent and longer antiplatelet therapy may be beneficial for this patient population.

## Introduction

In the clinical trial settings, heterogenous definitions of complex PCI have been applied across numerous studies in tailoring the duration of DAPT [1–5], resulting in a variety of outcome assessments reported in previous studies that limits the interpretation and generalizability of study results [6]. Since its initial introduction, although PCI complexity has been recognized as a contributor to future stent-driven ischemic events, several studies have examined the relationship of complex PCI with future bleeding events, reporting inconsistent results, in which some have confirmed its predictive value [4, 5] and some have not [1–3]. Against this background, the 2017 ESC dual antiplatelet therapy (DAPT) guidelines has been proposed to standardize the definition of high thrombotic risk (HTR) criteria of stent-driven recurrent ischemic events, including a composite of clinical (chronic kidney disease [CKD] and prior stent thrombosis on adequate antiplatelet therapy) and procedural characteristics (diffuse multivessel disease in diabetic patients, at least three stents implanted, at least three lesions treated, bifurcation with two stents implanted, total stent length > 60 mm, treatment of chronic total occlusion (CTO), or stenting of the last remaining coronary artery) [7]. Given these high-risk characteristics were mainly derived from previously published reports, data on the applicability of DAPT guideline-endorsed HTR criteria in the real-world practice is scare, especially in East Asian patients.

In this context, patients with HTR features may benefit from a longer duration of DAPT regimen to reduce risk of recurrent ischemic events [1]. However, concomitant high bleeding risk (HBR) may be present, making its benefits offset at least in part in these patients. It is known that patients at HBR remain at increased risk of both ischemic and hemorrhagic events after PCI. To date, HBR patients had not been well defined and the eligibility criteria of HBR patients were different among contemporary clinical trials [8–10]. Although several bleeding prediction scores are currently available to estimate the bleeding risks of the individual patient [11–14], they have moderate accuracy for predicting bleeding, with an average C statistics about 0.7. With this in mind, the Academic Research Consortium for HBR (ARC-HBR) developed a consensus definition of patients at HBR based on review of the available evidence in clinical trials [15]. Considering the mutual role and possibly competing role of HTR and ARC-HBR features on outcomes, whether DAPT guideline-endorsed HTR features exert similar or differential impact on the long-term occurrence of adverse events among patients with and without ARC-HBR after PCI in the real-world setting has not been well studied. Therefore, the purpose of this study was to (1) assess the ability of ESC guideline-endorsed HTR criteria to stratify ischemic and bleeding risk, and (2) examine whether ARC-HBR affects the association between HTR features and clinical outcomes differently using prospective data from an all-comers population receiving PCI with drug-eluting stents (DES).

## Methods

### Patient population

A total of 10,724 consecutive patients undergoing PCI at Fuwai Hospital (National Center for Cardiovascular Diseases, Beijing, China) were prospectively entered into the Fuwai PCI Registry (January 2013 to December 2013). For the present study, exclusion criteria were treatment by balloon angioplasty alone without stent placement, implantation of bioresorbable scaffolds or bare-metal stents, and unavailability of guideline-endorsed high-risk features for ischemic events at index PCI. Finally, 10,167 patients were selected for this analysis. The study was conducted based on the principles of the Declaration of Helsinki, and its protocol was approved by the hospital’s ethical review board (Fuwai Hospital & National Center for Cardiovascular Diseases, Beijing, China). All patients provided written informed consent for prospective follow-up before the intervention. Demographic and clinical characteristics, angiographic and procedural information, and follow-up data were systematically and prospectively collected in our dedicated PCI registry by independent research personnel. Details of the measurements and biochemical analysis are contained in the supplementary material method.

### Procedures and patient follow-up

The PCI procedure and best available medical therapy were performed in accordance with the current procedural guidelines [16, 17]. Detailed information on procedures is shown in the supplementary material method. After index PCI, patients were followed up at 1, 6, and 12 months and annually thereafter. Follow-up data were collected through medical records, telephone communications, or face-to-face interviews after hospital discharge by well-trained cardiologists who were blind to the purpose of the present study, until death occurred or up to the last day of the follow-up period. Patients were advised to return for coronary angiography if indications of ischemic events occurred. For patients treated for adverse events at other medical institutions, external medical records, discharge letters, and coronary angiography documentation were systematically collected and reviewed. The median follow-up duration was 29 months (interquartile range [IQR]: 26.5 to 31.1 months).

### Definitions and clinical outcomes

HTR criteria endorsed by 2017 ESC DAPT guidelines in the present study were defined with at least 1 of the following characteristics: diffuse (lesion length ≥ 20 mm) multivessel disease in diabetic patients, CKD (estimated glomerular filtration rate [eGFR] < 60 mL/min), at least three stents implanted, at least three lesions treated, bifurcation with two stents implanted, total stent length > 60 mm, or treatment of CTO. Patients are considered to be at HBR if at least 1 major or 2 minor criteria are met. In the present analysis, we modified the ARC-HBR definitions because several major and minor ARC-HBR criteria were not exactly captured in our registry. Therefore, those patients with at least one major criterion such as oral anticoagulation at discharge, severe CKD (eGFR< 30 ml/min), severe anemia (hemoglobin< 11 g/dL), thrombocytopenia (platelet count< 100 × 10^9^/L), previous stroke (≤12 months), and those with ≥2 minor criteria such as age ≥ 75 years, moderate CKD (eGFR 30–59 mL/min), or mild anemia (hemoglobin 11–12.9 g/dL for men and 11–11.9 g/dL for women) were classified as the HBR group. The primary ischemic outcome was major adverse cardiac event (MACE), defined as a composite of cardiac death, myocardial infarction (MI), target vessel revascularization (TVR), or definite/probable stent thrombosis (ST). The primary bleeding outcome was clinically relevant bleeding, defined as the Bleeding Academic Research Consortium (BARC) type 2, 3 or 5 bleeding [18]. Device oriented composite endpoints (DOCE) was defined as a composite of cardiac death, target-vessel (TV) MI, and target lesion revascularization (TLR). Detailed information on secondary outcomes and endpoint definitions are presented in the supplementary material method.

### Statistical analysis

Continuous variables are expressed as mean ± SD or median (interquartile range) and compared with the Student’s t-test or the Mann-Whiney U test, respectively. Categorical data are reported as numbers and percentages, and were compared using chi-square or Fisher’s exact test as appropriate. Cumulative event rates for ischemic and bleeding events were constructed using Kaplan-Meier method among those with and without HTR features and after substratifying all subjects by both HTR and HBR. Event rates were compared across groups using the log-rank test. Furthermore, the cumulative incidences of primary ischemic and bleeding outcomes were assessed according to the number of ESC-HTR criteria (0, 1 to 2, and ≥ 3 high-risk characteristics). The adjusted effect of “HTR features” on adverse events was estimated with multivariable Cox proportional hazard regression model, including “HTR features” as either a categorical or a continuous (per increase in number of HTR features) covariate in the Cox model, The covariates included in the model were age, sex, current smoking, hyperlipidemia, hypertension, acute coronary syndrome (ACS), left ventricular ejection fraction, peripheral artery disease, previous MI, previous revascularization (PCI and/or coronary artery bypass graft [CABG]), hemoglobin, white blood cell count, platelet count, type of DES, and duration of DAPT. Additionally, to evaluate the effect of the individual HTR features components on primary ischemic and bleeding outcomes, each was included as a separate predictor in the multivariable Cox model. Forming interaction testing was performed between HTR features and ARC-HBR on both ischemic and bleeding outcomes. All tests were two-sided and a P-value of < 0.05 was considered to be statistically significant. All analyses were performed with SPSS version 22.0 (SPSS Inc., Chicago, Illinois, USA).

## Results

### Clinical and procedural characteristics

A total of 10,167 patients were enrolled in Fuwai PCI registry and were included in the analyses. Of note, 4430 (43.6%) had at least 1 ESC-endorsed HTR criteria, and 5737 patients (56.4%) were considered to have no HTR features. In brief, patients at HTR features were more likely to be elderly with a high prevalence of common cardiovascular risk factors such as diabetes mellitus, hypertension, and hyperlipidemia; and had a higher proportion of stable CAD as an indication for PCI, previous MI, stroke, and CABG (Table 1). The ARC-HBR was more frequently occurred in patients with HTR than in patients without HTR (18.9% vs. 12.2%; *P* < 0.001). There were higher PARIS thrombotic (2.83 ± 1.82 vs. 2.27 ± 1.52; *P* < 0.001) and bleeding risk scores (3.86 ± 2.20 vs. 3.59 ± 1.97; *P* < 0.001), and longer duration of DAPT in ESC-HTR criteria group. During the index PCI, lesions were more complex among patients with HTR features, with more rates of left main or 3-vessel disease, heavy calcification, and thrombotic lesion and type B2/C lesions (Table 2). Moreover, patients with HTR features more frequently received glycoprotein IIb/IIIa inhibitors during the index procedure and transfemoral intervention, and intravascular ultrasound was more often used to guide the procedure in these patients. The overlap of DAPT guideline-endorsed HTR criteria is summarized in online Table 1. At least three stents implanted, at least three lesions treated and diffuse multivessel disease in diabetic patients frequently overlapped with other high-risk procedural characteristics.

**Table 1:** Baseline characteristics according to high thrombotic risk features

	HTR features (*n* = 4430)	Non-HTR features (*n* = 5018)	*P* value
Age, yrs	59.21 ± 10.27	57.64 ± 10.19	< 0.001
Age ≥ 75 years	338 (7.6)	293 (5.1)	< 0.001
Male	3396 (76.7)	4445 (77.5)	0.329
Body mass index, kg/m^2^	26.04 ± 3.17	25.84 ± 3.19	0.002
Hypertension	3027 (68.3)	3514 (61.3)	< 0.001
Diabetes mellitus	2073 (46.8)	969 (16.9)	< 0.001
Hyperlipidemia	3042 (68.7)	3795 (66.1)	0.007
eGFR	92.48 ± 20.66	97.06 ± 16.37	< 0.001
Moderate CKD (eGFR ≥ 30, < 60 ml/min/1.73m^2^)	353 (8.0)	0 (0.0)	< 0.001
Severe CKD (eGFR < 30 ml/min/1.73m^2^)	7 (0.2)	0 (0.0)	0.003
Current smoker	2509 (56.6)	3305 (57.6)	0.326
Previous myocardial infarction	940 (21.2)	980 (17.1)	< 0.001
Previous PCI	1007 (22.7)	1414 (24.6)	0.025
Previous CABG	219 (4.9)	184 (3.2)	< 0.001
Previous stroke	553 (12.5)	527 (9.2)	< 0.001
Peripheral arterial disease	147 (3.3)	120 (2.1)	< 0.001
LVEF, %	62.37 ± 7.54	63.20 ± 6.97	< 0.001
Indication			
Stable CAD	1905 (43.0)	2168 (37.8)	< 0.001
ACS	2525 (57.0)	3569 (62.2)	< 0.001
UA/NSTEMI	1980 (44.7)	2776 (48.4)	< 0.001
STEMI	545 (12.3)	793 (13.8)	0.025
Hemoglobin, g/dL	14.22 ± 1.57	14.36 ± 1.50	< 0.001
Severe anemia (Hb<11 g/dL)	98 (2.2)	80 (1.4)	0.002
Mild anemia (Hb: 11.0-12.9 g/dL for males or 11.0-11.9 g/dL for females)	461 (10.4)	479 (8.3)	< 0.001
Platelet count, 10^9^/L	204.63 ± 54.15	206.50 ± 56.27	0.090
Platelet<100×10^9^/L	44 (1.0)	49 (0.9)	0.465
White blood cell count, 10^9^/L	6.81 ± 1.64	6.69 ± 1.71	0.001
PARIS thrombotic risk score	2.83 ± 1.82	2.27 ± 1.52	< 0.001
PARIS bleeding risk score	3.86 ± 2.20	3.59 ± 1.97	< 0.001
PRECISE-DAPT score	11.54 ± 9.25	9.85 ± 7.77	< 0.001
Oral anticoagulation therapy	6 (0.1)	12 (0.2)	0.381
Duration of DAPT, days	576.49 ± 207.78	562.37 ± 208.25	0.001

Values are mean ± SD for continuous variables, and n (%) for categorical variables. *ACS* acute coronary syndrome(s), *CAD* coronary artery disease, *CKD* chronic kidney disease, *CABG* coronary artery bypass grafting, *DAPT* dual antiplatelet therapy, *HTR* high thrombotic risk, *LVEF* left ventricular ejection fraction, *MI* myocardial infarction, *NSTEMI* non-ST-segment elevation myocardial infarction, *PAD* peripheral artery disease, *PCI* percutaneous coronary intervention, *PARIS* Patterns of Non-Adherence to Anti-Platelet Regimen in Stented Patients, *STEMI* ST-segment elevation myocardial infarction, *UA* unstable angina

**Table 2:** Procedural characteristics according to high thrombotic risk features

	HTR features (n = 4430)	Non-HTR features (n = 5018)	*P* value
Left main or 3-vessel disease	2682 (60.5)	1841 (32.1)	< 0.001
Target vessel			
Left main artery	217 (4.9)	51 (0.9)	< 0.001
Left anterior descending artery	3760 (84.9)	5415 (94.4)	< 0.001
Left circumflex artery	1343 (30.3)	465 (8.1)	< 0.001
Right coronary artery	1481 (33.4)	394 (6.9)	< 0.001
Bypass graft	11 (0.2)	6 (0.1)	0.079
Number of lesion treated			< 0.001
1	1976 (44.6)	4802 (83.7)	
2	1721 (38.8)	935 (16.3)	
≥ 3	733 (38.8)	0 (0.0)	
Number of stents implanted	2.27 ± 1.00	1.34 ± 0.48	< 0.001
Total lesion length, mm	57.16 ± 30.18	24.98 ± 12.38	< 0.001
Total stent length, mm	60.76 ± 28.78	28.42 ± 12.20	< 0.001
Total stent length > 60 mm	2052 (46.3)	0 (0.0)	< 0.001
Mean stent diameter, mm	2.91 ± 30.53	3.09 ± 0.57	< 0.001
Diffuse multivessel disease in diabetic patients	1882 (42.5)	0 (0.0)	< 0.001
Bifurcation with two stents implanted	428 (9.7)	0 (0.0)	< 0.001
Treatment of chronic total occlusion	836 (18.9)	0 (0.0)	< 0.001
In-stent restenosis lesion	201 (4.5)	246 (4.3)	0.543
Heavy calcified lesion	229 (5.2)	112 (2.0)	< 0.001
Thrombotic lesion	194 (4.4)	201 (3.5)	0.023
Type B2 or C lesion	3987 (90.0)	3825 (66.7)	< 0.001
SYNTAX score	14.75 ± 8.40	9.23 ± 6.79	< 0.001
Glycoprotein IIb/IIIa use	885 (20.0)	764 (13.3)	< 0.001
Type of DES implanted			0.703
Early-generation DES	453 (10.2)	600 (10.5)	
New-generation DES	3977 (89.8)	5137 (89.5)	
Radial approach	3996 (90.2)	5275 (91.9)	0.002
Use of intravascular ultrasound	338 (7.6)	212 (3.7)	< 0.001

Values are mean ± SD for continuous variables, and n (%) for categorical variables. *DES* Drug eluting stents

### Clinical outcomes according to DAPT guideline-endorsed HTR features

**Fig. 1:**
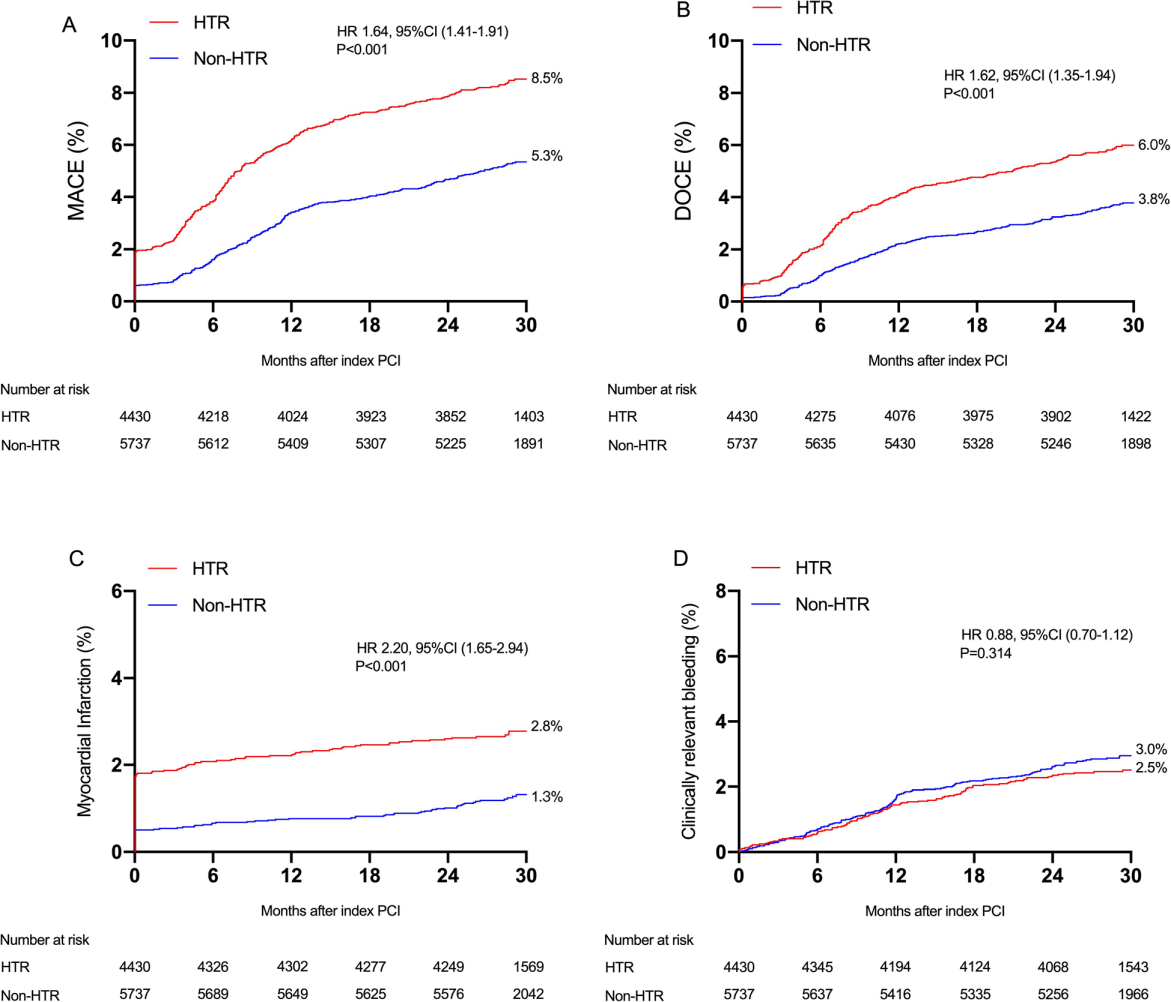
Kaplan-Meier event rates for the ischemic and bleeding outcomes according to ESC guideline-endorsed HTR status. Cumulative incidence of major adverse cardiac events (MACE) (**a**), device-oriented composite endpoint (DOCE) (**b**), myocardial infarction (**c**), clinically relevant bleeding (**d**). Red lines indicate patients with HTR. Blue lines indicate patients with non-HTR. HTR = high thrombotic risk; CI = confidence interval; HR = hazard ratio

At least 1 year’s follow-up were available for 10,117 (99.5%) with a median follow-up period of 29 months (IQR: 26 to 31 months). Patients presenting with ESC-HTR criteria for ischemic events had significantly higher rates of MACE (8.4% vs. 5.2%, *P* < 0.001), DOCE (5.8% vs. 3.7%, *P* < 0.001), cardiac death (0.9% vs. 0.5%, *P* < 0.001), MI (2.8% vs. 1.3%, *P* < 0.001), definite/probable ST (1.1% vs. 0.4%, *P* < 0.001), TVR (5.8% vs. 3.9%, *P* < 0.001) in comparison with patients with non-HTR criteria, but similar rate of clinically relevant bleeding (2.6% vs. 2.9%, *P* = 0.314) (Table 3 and Fig. 1). After controlling for potential confounders, HTR features were independently associated with increased hazards of MACE (adjusted hazard ratio [HR]: 1.56, 95% CI: 1.34–1.82; *P* < 0.001) (Table 3). Similarly, the risk of DOCE (HR_adjust_: 1.52 [1.27–1.83]), cardiac death (HR_adjust_: 1.85 [1.13–3.03]), MI (HR_adjust_: 2.05 [1.53–2.75]), TVR (HR_adjust_: 1.47 [1.22–1.76]), definite/probable ST (HR_adjust_: 2.47 [1.49–4.10]), stroke (HR_adjust_: 1.47 [1.08–2.01]) were also significantly higher in the HTR features group. The risk of clinically relevant bleeding was comparable between 2 groups (HR_adjust_: 0.85, 95% CI: 0.66–1.08; *P* = 0.174).

**Table 3:** Unadjusted and adjusted clinical outcomes according to HTR

			Unadjusted		Multivariable-Adjusted	
	HTR (n = 4430)	Non-HTR (n = 5737)	HR (95% CI)	*P* value	HR (95% CI)	*P* value
Major adverse cardiac event^a^	371 (8.4)	301 (5.2)	1.64 (1.41–1.91)	< 0.001	1.56 (1.34–1.82)	< 0.001
Device-oriented composite endpoint^b^	258 (5.8)	212 (3.7)	1.62 (1.35–1.94)	< 0.001	1.52 (1.27–1.83)	< 0.001
All-cause death	68 (1.5)	66 (1.2)	1.34 (0.95–1.88)	0.093	1.33 (0.94–1.89)	0.112
Cardiac death	42 (0.9)	30 (0.5)	1.82 (1.14–2.90)	0.013	1.85 (1.13–3.03)	0.014
Myocardial infarction	123 (2.8)	73 (1.3)	2.20 (1.65–2.94)	< 0.001	2.05 (1.53–2.75)	< 0.001
Target vessel Myocardial infarction	58 (1.3)	31 (0.5)	2.45 (1.59–3.80)	< 0.001	2.18 (1.40–3.39)	0.001
Any revascularization	463 (10.5)	424 (7.4)	1.45 (1.27–1.66)	< 0.001	1.42 (1.24–1.62)	< 0.001
Target vessel revascularization	257 (5.8)	226 (3.9)	1.51 (1.26–1.80)	< 0.001	1.47 (1.22–1.76)	< 0.001
Target lesion revascularization	200 (4.5)	172 (3.0)	1.54 (1.26–1.89)	< 0.001	1.50 (1.22–1.84)	< 0.001
Definite or probable stent thrombosis	48 (1.1)	23 (0.4)	2.72 (1.65–4.46)	< 0.001	2.47 (1.49–4.10)	< 0.001
Stroke	92 (2.1)	74 (1.3)	1.61 (1.18–2.18)	0.002	1.47 (1.08–2.01)	0.015
Any bleeding	293 (6.6)	403 (7.0)	0.94 (0.81–1.09)	0.432	0.91 (0.78–1.06)	0.242
Clinically relevant bleeding^c^	113 (2.6)	165 (2.9)	0.88 (0.70–1.12)	0.314	0.85 (0.66–1.08)	0.174

Values are number of events (%) unless otherwise indicated. The adjusted risk of adverse events after HTR versus non-HTR was assessed using a multivariable Cox proportional hazards regression adjusted for age, sex, current smoking, hyperlipidemia, hypertension, acute coronary syndrome, left ventricular ejection fraction, peripheral artery disease, previous myocardial infarction, previous revascularization (percutaneous coronary intervention and/or coronary artery bypass graft), hemoglobin, white blood cell count, platelet count, type of DES, and duration of DAPT. *CI* Confidence interval, *HR* Hazard ratio, *HTR* High thrombotic risk; other abbreviations as in Table 1 and Table 2

^a^ Major adverse cardiac events was defined as the composite of cardiac death, myocardial infarction, target vessel revascularization or definite/probable stent thrombosis

^b^ Device-oriented composite endpoint (DOCE) was defined as the composite of cardiac death, target-vessel myocardial infarction, and target lesion revascularization

^c^ Clinically relevant bleeding was defined as BARC type 2, 3, or 5 bleeding

**Fig. 2:**
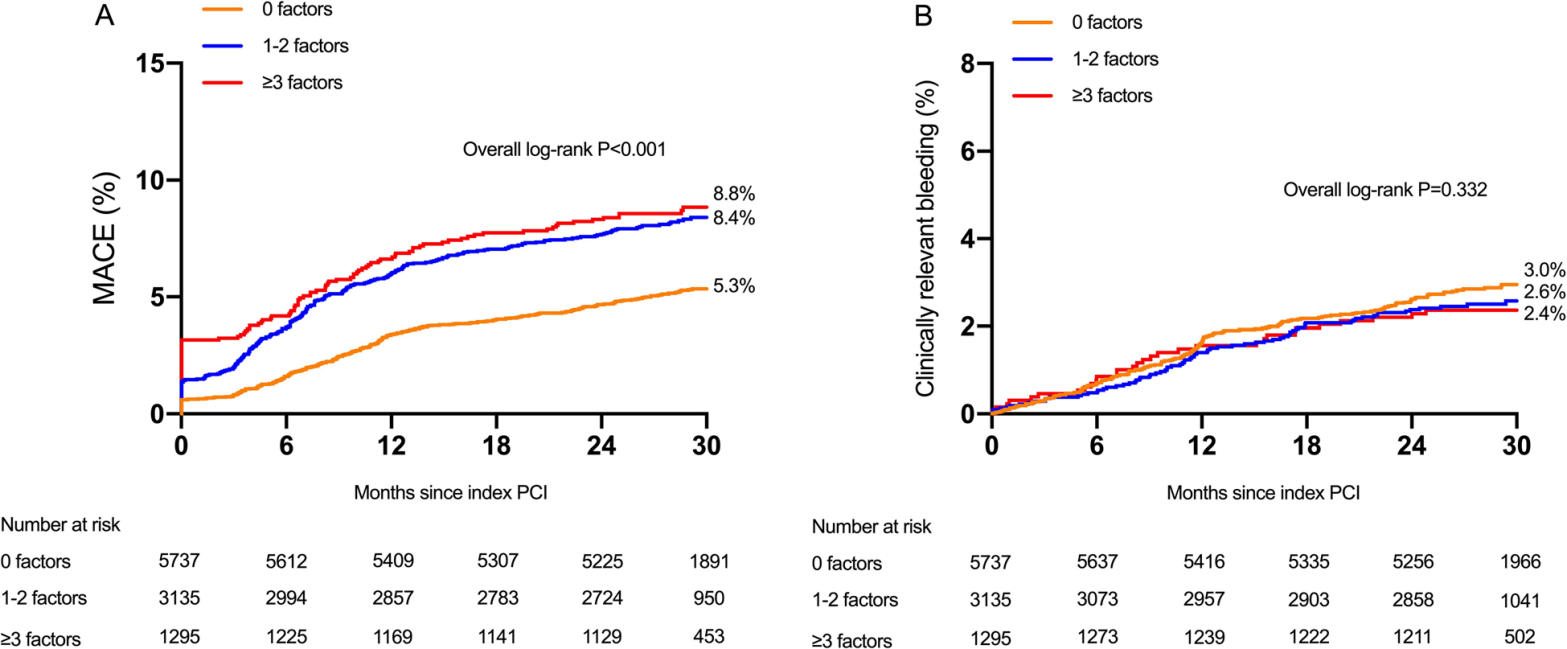
Kaplan-Meier event rates for MACE and clinically relevant bleeding stratified by the number of HTR features. Cumulative incidence of major adverse cardiac events (MACE) (**a**) and clinically relevant bleeding (**b**) stratified by the number of HTR features. HTR = high thrombotic risk

By including ESC-endorsed HTR criteria as a continuous variable (per number of high-risk features) within the same multivariable models, the risk of adverse ischemic events tended to be greater as the number of high-risk procedural characteristics increased (per number of high-risk variables increase: for MACE, HR_adjust_: 1.17, 95% CI: 1.11–1.23; P_trend_ < 0.001; for DOCE, HR_adjust_: 1.14, 95% CI: 1.07–1.22; P_trend_ < 0.001; for cardiac death, HR_adjust_: 1.24, 95% CI: 1.03–1.48; P_trend_ = 0.021). Of note, per number HTR criteria increase was not associated with greater risk of clinically relevant bleeding (HR_adjust_: 0.92, 95% CI: 0.83–1.02; P_trend_ = 0.107). As shown in Fig. 2, there was a gradual risk increase for MACE (0: 5.2%; 1 to 2: 8.2%; and ≥ 3: 8.8%; *p* < 0.001) as a function of the number of high-risk features. In constrast, there was a numerically gradual risk decrease for clinically relevant bleeding (0: 2.9%; 1 to 2: 2.6%; ≥3: 2.5%; *P* = 0.332) as the number of HTR features increased. Adjusted risk of each component of HTR features for MACE and clinically relevant bleeding is illustrated in online Tables 2 and 3. Specifically, 3 stents implanted (HR_adjust_: 1.37; 95% CI: 1.05 to 1.80), and ≥ 3 lesions treated (HR_adjust_: 1.30; 95% CI: 1.00 to 1.70) emerged as independent predictors for MACE, while each component of HTR feature was not related to an increased risk of clinically relevant bleeding.

### Clinical outcomes according to HTR features and ARC-HBR

Notably, subjects with ARC-HBR had significantly worse clinical outcomes compared with subjects without ARC-HBR. Subjects with ARC-HBR had significantly higher rates of all-cause death, MI, definite or probable ST, stroke, and clinically relevant bleeding (Online Table 4).

**Table 4:** Adjusted risk for adverse cardiac events according to HTR features in patients with and without ARC-HBR

	Non-ARC-HBR			ARC-HBR			
	HTR(n = 3591)	Non-HTR (n = 5038)	Adjusted HR (95% CI)	HTR features (n = 839)	Non-HTR features (n = 699)	Adjusted HR (95% CI)	*P* value for interaction
Major adverse cardiac event	297 (8.3)	259 (5.1)	1.59 (1.34–1.88)	74 (8.8)	42 (6.0)	1.46 (0.99–2.16)	0.543
Device-oriented composite endpoint	205 (5.7)	182 (3.6)	1.54 (1.26–1.89)	53 (6.3)	30 (4.3)	1.43 (0.90–2.26)	0.614
All-cause death	45 (1.3)	49 (1.0)	1.40 (0.92–2.13)	23 (2.7)	17 (2.4)	0.94 (0.49–1.83)	0.397
Cardiac death	25 (0.7)	23 (0.5)	1.66 (0.91–3.02)	17 (2.0)	7 (1.0)	1.69 (0.64–4.44)	0.895
Myocardial infarction	96 (2.7)	57 (1.1)	2.31 (1.66–3.22)	27 (3.2)	16 (2.3)	1.26 (0.67–2.37)	0.102
Target vessel revascularization	213 (5.9)	199 (3.9)	1.49 (1.23–1.81)	44 (5.2)	27 (3.9)	1.39 (0.85–2.27)	0.690
Definite or probable stent thrombosis	34 (0.9)	15 (0.3)	3.20 (1.73–5.91)	14 (1.7)	8 (1.1)	1.28 (0.53–3.10)	0.072
Stroke	55 (1.5)	60 (1.2)	1.23 (0.85–1.78)	37 (4.4)	14 (2.0)	2.08 (1.11–3.88)	0.194
Clinically relevant bleeding	84 (2.3)	131 (2.6)	0.89 (0.68–1.18)	29 (3.5)	34 (4.9)	0.63 (0.38–1.04)	0.269

Values are number of events (%) unless otherwise indicated. The adjusted risk of adverse events after HTR versus non-HTR was assessed using a multivariable Cox proportional hazards regression adjusted for age, sex, current smoking, hyperlipidemia, hypertension, acute coronary syndrome, left ventricular ejection fraction, peripheral artery disease, previous myocardial infarction, previous revascularization (percutaneous coronary intervention and/or coronary artery bypass graft), hemoglobin, white blood cell count, platelet count, type of DES, and duration of DAPT

*ARC* Academic Research Consortium, *HBR* High bleeding risk, other abbreviations as in Table 1 and Table 2

**Fig. 3:**
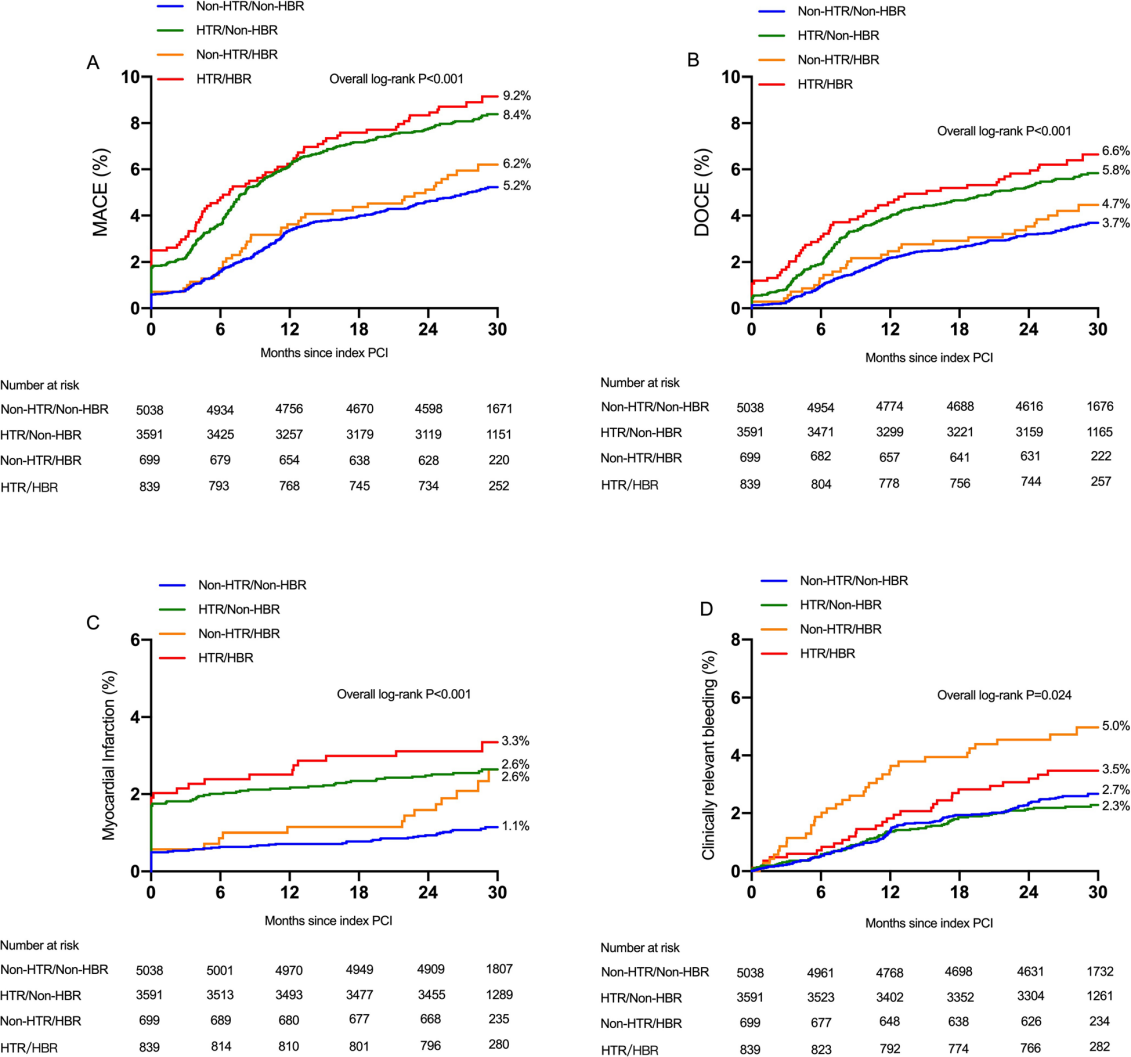
Kaplan-Meier event rates for the ischemic and bleeding outcomes according to according to ESC guideline-endorsed HTR and ARC-HBR status. Cumulative incidence of major adverse cardiac events (MACE) (**a**), device-oriented composite endpoint (DOCE) (**b**), myocardial infarction (**c**), clinically relevant bleeding (**d**). Red lines indicate patients with both HTR and HBR. Green lines indicate patients with non-HTR and HBR. Orange lines indicte patients with HTR and non-HBR. Blue lines indicate patients with both non-HTR and non-HBR. HTR = high thrombotic risk; HBR = high bleeding risk

In contrast, ischemic event rates were higher among those with versus without HTR features, but bleeding rates were lower among those with HPR irrespective of ARC-HBR status. As shown in Fig. 2, the rates of MACE among those without HTR or HBR, HBR alone, HTR alone, and both HTR and HBR were 5.2, 6.2, 8.4 and 9.2%, respectively (Fig. 3a; *P* < 0.001). Similar patterns of higher risk were observed for DOCE (Fig. 3b) and MI (Fig. 3c). The rate of clinically relevant bleeding was higher among subjects with HBR, although HTR showed nonstatistically significant low rates of major bleeding (*P* = 0.024). Clinically relevant bleeding rates across these same 4 groups were 2.7, 5.0, 2.3, 4.5%, respectively (Fig. 3d).

Adjusted HRs for ischemic and bleeding events associated with HTR features and stratified by the presence or absence of ARC-HBR are shown in Table 4. The HRs of any end point were similar in the direction and magnitude among the HBR and non-HBR groups with no evidence of statistical interaction (all P_interaction_ > 0.05), suggesting a consistent effect within ESC guideline-endorsed HTR features. There was no significant interaction (adjusted P_interaction_ = 0.543) in the adverse effect of HTR versus non-HTR features for MACE between patients with HBR (HR_adj_: 1.46, 95% CI: 0.99–2.16) and non-HBR (HR_adjust_: 1.59, 95% CI: 1.34–1.88). The unadjusted rates of DOCE, cardiac death, MI, definite/probable ST, TVR, stroke were higher in HBR subjects with HTR in relative to HBR subjects without HTR; however, after multivariable adjustment, the HRs were not significantly different due to analysis of subjects with HBR is limited by small sizes. It was worthy of noting that the risk of clinically relevant bleeding associated with HTR features was not increased in participants with HBR (HR_adjust_: 0.63, 95% CI: 0.38–1.04) and those without HBR (HR_adjust_: 0.89, 95% CI: 0.68–1.18; P_interaction_ = 0.269). Moreover, the effect of HTR versus non-HTR features on MACE (HR_adjust_: 1.71 [1.34–2.18] with stable CAD and HR_adjust_: 1.46 [1.19–1.78] with ACS, P_interaction_ = 0.330) and clinically relevant bleeding (HR_adjust_: 0.99 [0.68–1.43] with stable CAD and HR_adjust_: 0.75 [0.54–1.03] with ACS, P_interaction_ = 0.351) was similar regardless of the patient presented with stable CAD or ACS.

## Discussion

This study was the first study to validate the DAPT guideline-endorsed HTR criteria and characterized the interplay between these HTR criteria, ARC-HBR status, and clinical outcomes in an all-comer patient population with unselected use of DES from a prospective real-world registry. The salient findings of the present analysis were as follows: (1) HTR criteria recommended by 2017 ESC DAPT guidelines successfully identified those patients with very HTR after PCI, who represented 44% of patients in this all-comers registry; (2) ECS-endorsed HTR criteria showed a significantly greater risk of MACE while maintaining a similar risk of clinically relevant bleeding. In addition, HTR was associated with lower risks of DOCE, as well as cardiac death, MI, any revascularization, ST, and stroke; (3) The independent impact of HTR features on thrombotic events was substantial and uniform irrespective of HBR status, and there was no an interaction between HTR features and HBR on clinically relevant bleeding, suggesting that intensified DAPT may be beneficial to patients at HTR features; (4) Individual HTR features, such as ≥3 stents implanted and ≥ 3 lesions treated, which were the angiographic subset most consistently and strongly associated with increased ischemic risk but not for bleeding, may be helpful to guide potent P2Y_12_ inhibitors or prolongation of DAPT.

The extent and complexity of CAD significantly affect the optimum invasive method for revascularization and strongly influence outcomes after PCI [19, 20]. In case of PCI as the preferred strategy, patients with coronary anatomic complexity and challenging subsets of lesions result in complex PCI procedures and at higher risk for adverse clinical events [21, 22]. Although patients who underwent complex PCI have consistently been reported to be associated with a higher incidence of subsequent ischemic events, the relative risks of complex PCI in terms of clinically relevant bleeding are a matter of debate [1–5]. Given that there have been no universal criteria of complex PCI with regard to angiographic and lesion-related features, leading to different outcome assessments reported in previous studies [6], the 2017 ESC DAPT guidelines proposed the concept of HTR features of stent-driven ischemic events, defined according to 9 clinical and procedural criteria, to identify patients who may receive more benefit from a longer period of DAPT [7]. In routine clinical practice, clinical decision-making on upfront DAPT duration and intensity after complex PCI warrants a simultaneous appraisal of both ischemic and bleeding complications. It is known that patients at HBR need careful evaluation owing to their high risk of thrombotic and bleeding complications when determining potency or duration of antithrombotic management [9, 23]; however, whether ESC-endorsed HTR features confer a similar or differential risk for ischemic and bleeding events among those with and without HBR has, to our knowledge, not yet been elucidated in all-comer patients cohort because HBR patients are mostly excluded from randomized controlled trials (RCT) of PCI [24, 25]. We, therefore, performed the present analysis that, with approximately 10,000 patients, represents one of the largest real-world population assessing the impact of ESC-HTR features on clinical outcomes after coronary DES implantation and whether this effect is influenced by HBR status.

This large single-center PCI cohort reflecting a real-world setting were coherent with those of prior findings using randomized trial data in that ESC-HTR features not only exerted an adverse impact on MACE and DOCE proportional to the number of HTR criteria present, but also on all individual endpoints including cardiac death, MI, definite/probable ST, and TVR [1, 2, 5]. On the other hand, the HTR criteria group did not experience a parallel increase in the risk of clinically relevant bleeding, as compared with those at non-HTR criteria. This was in line with two large analyses showing comparable bleeding risks between complex and non-complex PCI groups [1, 2]. In contrast to our findings, an all-comer sample of Bern PCI registry described that patients having at least 1 of high-risk features of stent-related recurrent ischemic events had a higher risk of bleeding (BARC 3–5) and ischemic (DOCE) events [26], making optimal duration and intensity of DAPT challenging for these patients. Several reasons might explain for the lack of a significant association noted in our study between HTR features and clinically relevant bleeding. First, the conflicting results may be attributable to differences in the proportions of each HTR component. Owing to the fact that CKD has emerged as a common contributor to both type of ischemic and bleeding complications [13, 27], the proportion of CKD that was markedly lower (4.0%) in our study than in Bern PCI registry (24.7%) might account for the similar risk of clinically relevant bleeding in HTR versus non-HTR features group in the present study. Second, considering that patients with diabetes or multivessel CAD represent an advanced state of atherosclerosis, with higher rates of in-hospital events, as well as recurrent atherothrombotic coronary events and death [28–31], diffuse multivessel disease in diabetic patients (18.5%) constituted the majority of our DAPT guideline-endorsed HTR features, thereby being positioned to clinical tendencies to ischemic events. Given the low number of patients with diffuse multivessel disease and diabetes (2.7%) in Bern PCI registry [26], it would be possible to detect a difference in the effect of HTR features on bleeding events. It should also be mentioned that high as many as 20% of our patients had undergone a PCI with total stent length > 60 mm (of note, the proportion of patients with long stent stenting in Bern PCI registry was 6.5%). Third, there is evidence demonstrating an increased risk of bleeding events in patients with baseline higher categories of bleeding risk prediction scores [32, 33]. Lower bleeding risk of our study patients, compared with Bern PCI registry (PRECISE-DAPT score: 10.6 ± 8.5 vs. 20.6 ± 13.3), may have partly explain difference in the risk of clinically relevant bleeding. In addition, differences in the intensity of DAPT (clopidogrel vs. more potent P2Y_12_ inhibitors) may, in part, relate to the discordant results. Indeed, Ueki et al. [26] showed that nearly 40% of patients involved used of more potent P2Y_12_ inhibitor such as ticagrelor and prasugrel, yet only clopidogrel was used as a P2Y_12_ inhibitor for DAPT in our study because potent P2Y12 receptor blockers such as ticagrelor or prasugrel were not available in China during the study period.

While individual patient risks of ischemia and bleeding are related to many common risk factors, little is known regarding the impact of HTR features and the risk of adverse events according to the underlying bleeding risk [34]. In the current analysis from the Fuwai PCI registry, we found no convincing evidence of an interaction between HTR features and ARC-HBR in regard to the risk of ischemic and bleeding outcomes. In other words, ARC-HBR further increases the risk of long-term adverse events after PCI of both ESC-HTR and non-ESC-HTR criteria to a comparable degree. Bleeding risk may not be increased to the same extent as ischemic risk in HTR patients, which sets the rationale for supporting that ESC-HTR features may be a useful parameter for risk stratification of patients with HBR after PCI. Given the higher risk of ischemic complications in patients with ESC-HTR characteristics, effective antiplatelet therapy may be particularly important for these patients. Consistent with this hypothesis, longer-term DAPT has recently been shown to be more effective in patients who underwent complex PCI [1].

### Study limitations

First, the study has an innate limitation regarding its observational nature with registry data. PCI procedures was determined at the discretion of the attending physician and might have been influenced by several factors such as underlying demographics, clinical presentation at admission, and physician’s preference. Despite the implementation of multivariable Cox regression analysis to adjust for potential confounding factors and minimize the bias from different baseline characteristics, residual confounding cannot be excluded. Second, although majority of ESC-endorsed HTR features were taken into account in this analysis, information on prior ST on adequate antiplatelet therapy and stenting of the last remaining coronary artery were not captured in Fuwai PCI dataset, thus limiting their applicability in our population and representing an important restriction. Furthermore, because Fuwai PCI registry was not designed to investigate the performance of ARC-HBR criteria, some ARC-HBR criteria were not applicable. Thus, the prevalence of HBR patients would have been underestimated in our study. Third, despite the sample size of this cohort, the analyses assessing the effect of HTR features on clinical outcomes in the HBR subgroup and the interaction testing for the effect of HTR features on clinical outcomes, stratified by presence or absence of HBR, are likely underpowered. Finally, all patients were treated with clopidogrel, although approximately 60% of patients enrolled presented with acute coronary syndromes, and ticagrelor had not yet been approved during the time of enrollment. Our findings warrant confirmation in larger samples treated with potent P2Y_12_ inhibitors.

## Conclusions

In this single-center, all-comers population treated with DES, HTR features recommended by 2017 ESC DAPT guidelines were independently associated with an increase in the risks of MACE and DOCE with no apparent clinically relevant bleeding liability. The impact of HTR features on ischemic events is consistent among patients with HBR without evidence of an excess hazard of clinically relevant bleeding. Our data suggest that ESC-endorsed HTR criteria was useful for stratifying post-PCI patients into risk strata for future ischemic events as well informing us that ensuring adequate platelet inhibition may be beneficial for reducing the risk of adverse cardiovascular outcomes in patients with HTR features.

## Supplementary information

**Additional file 1.**

## Data Availability

The datasets generated and/or analyzed during the current study are available from the corresponding author on reasonable request.

## Abbreviations

ARC-HBR: Academic research consortium for high bleeding risk
ACS: Acute coronary syndrome
BARC: Bleeding academic research consortium
CKD: Chronic kidney disease
CTO: Chronic total occlusion
CAD: Coronary artery disease
CI: Confidence interval
CABG: Coronary artery bypass graft
DAPT: Dual antiplatelet therapy
DES: Drug-eluting stents
DOCE: Device oriented composite endpoints
eGFR: Estimated glomerular filtration rate
HTR: High thrombotic risk
HBR: High bleeding risk
HR: Hazard ratio
IQR: Interquartile range
MI: Myocardial infarction
MACE: Major adverse cardiac event
PCI: Percutaneous coronary intervention
RCT: Randomized controlled trials
ST: Stent thrombosis
TVR: Target vessel revascularization
TLR: Target lesion revascularization
