# Supplementary material for: Contribution of ESC DAPT guideline-endorsed high thrombotic risk features to long-term clinical outcomes among patients with and without high bleeding risk after PCI
Source: BMC Cardiovasc Disord. 2020 Jul 1;20:313. doi: 10.1186/s12872-020-01600-3 (PMC7329419; doi:10.1186/s12872-020-01600-3)
Supplement: Supplementary file 1 — Additional file 1. [file 12872_2020_1600_MOESM1_ESM.docx]

**Supplementary Material**

**Title:** Contribution of ESC DAPT guideline-endorsed high thrombotic risk features to long-term clinical outcomes among patients with and without high bleeding risk after PCI

**Supplementary methods**

**Clinical and laboratory analysis**

At baseline, during a personal interview, the information on demographic factors, medical history, medication use, and personal health habits was collected from each subject. Body mass index (BMI) was calculated as weight per height squared (kg/m^2^). The subjects were reminded of keeping an overnight fasting with 12 hours before the investigation. Venous blood specimens were designed to be obtained for assessing total cholesterol (TC), triglyceride (TG), low-density lipoprotein cholesterol (LDL-C), high-density lipoprotein cholesterol (HDL-C), fasting plasma glucose (FPG), hemoglobin A1c (HbA1c), and serum creatinine by automatic Biochemistry analyzer (Hitachi 7150, Tokyo, Japan) and enzymatic assay. The definition of hypertension was established as blood pressure (BP) level of at least 140/90 mmHg, individuals who were on antihypertensive medications or a prior diagnosis of hypertension. Diabetes was recognized in patients with a fasting glucose ≥7.0 mmol/L, the 2-h plasma glucose of the oral glucose tolerance test ≥11.0 mmol/L, HbA1c>6.5%, or current use of hypoglycemic drugs or insulin. Chronic kidney disease (CKD) is defined as glomerular filtration rate<60 ml/min/1.73 m^2^ using the CKD-Epidemiology collaboration definition [1].

**Procedures**

All patients received a loading dose of aspirin (300 mg) and clopidogrel 300 to 600 mg before PCI unless they had previously received these antiplatelet medications. Unfractionated heparin at a dose of 100 IU/kg was administered during the procedure to maintain an activated clotting time of 250 to 300s. The access site, revascularization treatment strategy, periprocedural use of glycoprotein IIb/IIIa receptor inhibitors, use of intravascular ultrasound, and selection of DES were left to the discretion of the operator. After the index procedure, aspirin at a dose of 100 mg once daily was continued indefinitely, and clopidogrel (75 mg once daily) was recommended for at least one year. New P2Y12 receptor inhibitors were not available during the study period in China. Optimal medications therapy, including β-blocker, renin-angiotensin system blockade, nitrates, calcium channel blocker, and statins were also recommended to all patients at the discretion of the responsible clinicians.

**Clinical outcomes definitions**

Other endpoints evaluated in the present study included all-cause death, cardiac death, MI, TV-MI, any repeat revascularization, TVR, TLR, definite/probable ST, stroke, any bleeding, and BARC type 3 or 5 bleeding. A clinical event committee consisting of 2 cardiologists (and a third in case of disagreement) adjudicated all events by using original source documents. All deaths were considered cardiac unless a definite non-cardiac cause could be established. MI was deﬁned as the presence of ischemic symptoms, electrocardiographic changes, or abnormal imaging findings consistent with myocardial ischemia in the setting of increased creatine kinase-myocardial band or troponin above the upper limit of normal in accordance with the universal deﬁnition [2]. Definite or probable stent thrombosis was defined according to the Academic Research Consortium (ARC) classification [3]. Revascularization was defined as repeat revascularization for ischemic symptoms and events driven by PCI or surgery of any vessel. Target lesion revascularization was deﬁned as any clinically indicated revascularization for a stenosis >50% within the stent or the 5-mm borders adjacent to the stent. Target vessel revascularization was deﬁned as repeat revascularization of any segment within the entire major coronary vessel, proximal and distal to a target lesion. Stroke was defined as any non-convulsive focal or global neurological deficit of abrupt onset lasting more than 24 h or leading to death, which was caused by ischemia or hemorrhage within the brain. Stroke was confirmed by a neurologist on the basis of imaging studies. Bleeding events were categorized on the basis of the Bleeding Academic Research Consortium (BARC) classifications. We classified BARC types 2, 3, or 5 bleeding as clinically relevant bleeding for the present analysis.

**References:**

[1] Levey AS, Stevens LA, Schmid CH, Zhang YL, Castro AF, 3rd, Feldman HI, et al. A new equation to estimate glomerular filtration rate. Annals of internal medicine. 2009;150:604-12.

[2] Thygesen K, Alpert JS, Jaffe AS, Simoons ML, Chaitman BR, White HD, et al. Third universal definition of myocardial infarction. Journal of the American College of Cardiology. 2012;60:1581-98.

[3] Cutlip DE, Windecker S, Mehran R, Boam A, Cohen DJ, van Es GA, et al. Clinical end points in coronary stent trials: a case for standardized definitions. Circulation. 2007;115:2344-51.

Online Table 1: Overlap between components of HTR features

|  | Diffuse multivessel disease in diabetic patients | Chronic kidney disease | ≥3 stents implanted | ≥3 lesions treated | Bifurcation with two stents implanted | Total stent length>60mm | Treatment of chronic total occlusion |
| --- | --- | --- | --- | --- | --- | --- | --- |
| Diffuse multivessel disease in diabetic patients (n=1882) |  | 97 (5.2%) | 726 (38.6%) | 230 (12.2%) | 92 (4.9%) | 636 (33.8%) | 212 (11.3%) |
| Chronic kidney disease (n=404) | 97 (24.0%) |  | 107 (26.5%) | 42 (10.4%) | 14 (3.5%) | 92 (22.8%) | 36 (8.9%) |
| ≥3 stents implanted (n=2385) | 726 (30.4%) | 107 (4.5%) |  | 598 (25.1%) | 262 (11.0%) | 1817 (76.2%) | 394 (16.5%) |
| ≥3 lesions treated (n=734) | 230 (31.3%) | 42 (5.7%) | 598 (81.5%) |  | 91 (12.4%) | 500 (68.1%) | 137 (18.7%) |
| Bifurcation with two stents implanted (n=428) | 92 (21.5%) | 14 (3.3%) | 262 (61.2%) | 163 (38.1%) |  | 163 (38.1%) | 39 (9.1%) |
| Total stent length>60mm (n=2052) | 636 (31.0%) | 92 (4.5%) | 1817 (76.2%) | 500 (24.4%) | 163 (7.9%) |  | 382 (18.6%) |
| Treatment of chronic total occlusion (n=836) | 212 (25.4%) | 36 (4.3%) | 394 (47.1%) | 137 (16.4%) | 39 (4.7%) | 382 (45.7%) |  |

Values are n (%). HTR = high thrombotic risk

Online Table 2: Effect of each HTR feature on MACE

|  | Univariate | | Multivariate model 1 | | Multivariate model 2 | |
| --- | --- | --- | --- | --- | --- | --- |
|  | HR (95% CI) | P value | Adjusted HR (95% CI) | P value | Adjusted HR (95% CI) | P value |
| Diffuse multivessel disease in diabetic patients | 1.30 (1.08-1.56) | 0.005 | 1.25 (1.04-1.50) | 0.017 | 1.16 (1.04-1.50) | 0.126 |
| Chronic kidney disease | 1.58 (1.15-2.18) | 0.005 | 1.31 (0.94-1.84) | 0.112 | 1.32 (0.94-1.85) | 0.105 |
| ≥3 stents implanted | 1.48 (1.26-1.75) | <0.001 | 1.49 (1.26-1.75) | <0.001 | 1.37 (1.05-1.80) | 0.022 |
| ≥3 lesions treated | 1.62 (1.27-2.06) | <0.001 | 1.62 (1.27-2.07) | <0.001 | 1.30 (1.00-1.70) | 0.055 |
| Bifurcation with 2 stents | 1.42 (1.03-1.97) | 0.033 | 1.45 (1.05-2.01) | 0.024 | 1.23 (0.88-1.72) | 0.224 |
| >60mm total stent length | 1.36 (1.14-1.62) | 0.001 | 1.36 (1.14-1.62) | 0.001 | 0.93 (0.70-1.22) | 0.590 |
| Chronic total occlusion | 1.42 (1.12-1.81) | 0.004 | 1.37 (1.08-1.75) | 0.011 | 1.23 (0.96-1.58) | 0.099 |

In the model 1, each HTR feature was adjusted by following variables. In the model 2, each HTR feature was adjusted by following variables and all components of the HTR criteria. Variables: age, sex, current smoking, hyperlipidemia, hypertension, acute coronary syndrome, left ventricular ejection fraction, peripheral artery disease, previous myocardial infarction, previous revascularization (percutaneous coronary intervention and/or coronary artery bypass graft), hemoglobin, platelet count, and type of DES. CI = confidence interval; HR = hazard ratio; HTR = high thrombotic risk; MACE = major adverse cardiac events.

Online Table 3: Effect of each HTR feature on clinically relevant bleeding

|  | Univariate | | Multivariate model 1 | | Multivariate model 2 | |
| --- | --- | --- | --- | --- | --- | --- |
|  | HR (95% CI) | P value | Adjusted HR (95% CI) | P value | Adjusted HR (95% CI) | P value |
| Diffuse multivessel disease in diabetic patients | 0.93 (0.69-1.27) | 0.661 | 0.88 (0.65-1.20) | 0.432 | 0.92 (0.67-1.27) | 0.618 |
| Chronic kidney disease | 1.42 (0.84-2.38) | 0.191 | 0.94 (0.55-1.62) | 0.825 | 0.95 (0.55-1.64) | 0.856 |
| ≥3 stents implanted | 0.79 (0.59-1.06) | 0.113 | 0.77 (0.57-1.04) | 0.085 | 0.69 (0.43-1.12) | 0.134 |
| ≥3 lesions treated | 0.77 (0.47-1.28) | 0.311 | 0.75 (0.45-1.25) | 0.268 | 0.87 (0.50-1.50) | 0.609 |
| Bifurcation with 2 stents | 0.93 (0.51-1.69) | 0.804 | 0.98 (0.54-1.79) | 0.947 | 1.12 (0.60-2.08) | 0.719 |
| >60mm total stent length | 0.90 (0.66-1.21) | 0.471 | 0.88 (0.65-1.19) | 0.396 | 1.27 (0.79-2.05) | 0.329 |
| Chronic total occlusion | 0.64 (0.38-1.12) | 0.091 | 0.65 (0.39-1.12) | 0.125 | 0.70 (0.41-1.19) | 0.183 |

In the model 1, each HTR feature was adjusted by following variables. In the model 2, each HTR feature was adjusted by following variables and all components of the HTR criteria. Variables: age, sex, current smoking, hyperlipidemia, hypertension, acute coronary syndrome, left ventricular ejection fraction, peripheral artery disease, previous myocardial infarction, previous revascularization (percutaneous coronary intervention and/or coronary artery bypass graft), hemoglobin, platelet count, and type of DES. CI = confidence interval; HR = hazard ratio; HTR = high thrombotic risk.

Online Table 4: Event rates in subjects with versus without ARC-HBR after coronary stenting

|  | ARC-HBR  (n=1538) | Non-ARC-HBR  (n=8629) | HR (95% CI) | P value |
| --- | --- | --- | --- | --- |
| All-cause death | 40 (2.6%) | 94 (1.1%) | 2.42 (1.67-3.50) | <0.001 |
| Cardiac death | 24 (1.6%) | 48 (0.6%) | 2.85 (1.75-4.65) | <0.001 |
| Myocardial infarction | 43 (2.8%) | 153 (1.8%) | 1.59 (1.13-2.23) | <0.001 |
| Target vessel revascularization | 71 (4.6%) | 412 (4.8%) | 0.98 (0.76-1.26) | 0.851 |
| Definite or probable stent thrombosis | 22 (1.4%) | 49 (0.6%) | 2.55 (1.54-4.21) | <0.001 |
| Stroke | 51 (3.3%) | 115 (1.3%) | 2.54 (1.82-3.53) | <0.001 |
| Clinically relevant bleeding | 63 (4.1%) | 215 (2.5%) | 1.67 (1.25-2.20) | <0.001 |

Values are number of events (%). ARC-HBR = Academic Research Consortium for High Bleeding Risk; other abbreviations as in Supplementary Table 2.
